# Molecularly defined sinonasal malignancies: an overview with focus on the current WHO classification and recently described provisional entities

**DOI:** 10.1007/s00428-024-03775-y

**Published:** 2024-03-16

**Authors:** Alena Skálová, Abbas Agaimy, Martina Bradova, Vincent Vander Poorten, Ehab Hanna, Orlando Guntinas-Lichius, Alessandro Franchi, Henrik Hellquist, Roderick H. W. Simpson, Fernando Lopéz, Sandra Nuyts, Carlos Chiesa-Estomba, Sweet Ping Ng, Akihiro Homma, Yong Teng, Ilmo Leivo, Alfio Ferlito

**Affiliations:** 1 Sikl’s Department of Pathology, Faculty of Medicine in Pilsen, Charles University, E. Benese 13, 305 99 Pilsen, Czech Republic; 2 Bioptic Laboratory, Ltd., Pilsen, Czech Republic; 3 Institute of Pathology, University Hospital Erlangen, Friedrich‐Alexander University Erlangen‐Nürnberg (FAU), Erlangen, Germany; 4 Department of Otorhinolaryngology-Head and Neck Surgery, Leuven Cancer Institute, University Hospitals Leuven, 3000 Louvain, Belgium; 5 Department of Oncology, Section Head and Neck Oncology, Leuven Cancer Institute, KU Leuven, Louvain, Belgium; 6 Department of Head & Neck Surgery, The University of Texas MD Anderson Cancer Center, Houston, TX USA; 7 Department of Otorhinolaryngology, Jena University Hospital, Jena, Germany; 8 Department of Translational Research, School of Medicine, University of Pisa, Pisa, Italy; 9 Faculty of Medicine and Biomedical Sciences (FMCB), Biomedical Center Research Institute (ABC-RI), University of Algarve, Faro, Portugal; 10 Department of Anatomical Pathology, University of Calgary, Calgary, AB Canada; 11 Department of Otolaryngology, ISPA, IUOPA, CIBERONC, Hospital Universitario Central de Asturias, University of Oviedo, Oviedo, Spain; 12 Laboratory of Experimental Radiotherapy, Department of Oncology, Leuven Cancer Institute, University Hospitals Leuven, 3000 Louvain, Belgium; 13 Department of Radiation Oncology, Leuven Cancer Institute, University Hospitals Leuven, 3000 Louvain, Belgium; 14 Department of Otorhinolaryngology-Head and Neck Surgery, Hospital Universitario Donostia, Donostia-San Sebastian, Guipuzkoa-Basque Country Spain; 15 Department of Radiation Oncology, Olivia Newton-John Cancer Wellness and Research Centre, Austin Health, Melbourne, Australia; 16 Department of Otolaryngology-Head and Neck Surgery, Faculty of Medicine, Graduate School of Medicine, Hokkaido University, Hokkaido, Japan; 17 Department of Hematology and Medical Oncology, Winship Cancer Institute, Emory University School of Medicine, Atlanta, GA USA; 18 Institute of Biomedicine, Pathology, University of Turku, Turku, Finland; 19 Coordinator of the International Head and Neck Scientific Group, Padua, Italy; 20 Department of Pathology, Turku University Hospital, Turku, Finland

**Keywords:** Sinonasal tumor, Sinonasal, Soft tissue, Head and neck, Molecular diagnostics, Next-generation sequencing

## Abstract

Classification of tumors of the head and neck has evolved in recent decades including a widespread application of molecular testing in tumors of the sinonasal tract, salivary glands, and soft tissues with a predilection for the head and neck. The availability of new molecular techniques has allowed for the definition of multiple novel tumor types unique to head and neck sites. Moreover, an expanding spectrum of immunohistochemical markers specific to genetic alterations facilitates rapid identification of diagnostic molecular abnormalities. As such, it is currently possible for head and neck pathologists to benefit from a molecularly defined tumor classification while making diagnoses that are still based largely on histopathology and immunohistochemistry. This review covers the principal molecular alterations in sinonasal malignancies, such as alterations in *DEK*, *AFF2*, *NUTM1*, *IDH1-2*, and *SWI/SNF* genes in particular, that are important from a practical standpoint for diagnosis, prognosis, and prediction of response to treatment.

## Introduction

Classification of head and neck neoplasms has improved in recent decades with the widespread application of molecular testing. Not only has molecular testing allowed for the definition of multiple novel tumor types unique to the head and neck, but it has also facilitated the recognition of ubiquitous tumors that commonly involve the head and neck. Molecular testing has illuminated the pathogenesis of well-established but previously enigmatic entities and clarified the relationships between various neoplasms. The current 5th edition of the *World Health Organization (WHO) Classification of Head and Neck Tumours* relies heavily on molecular data to support the inclusion of several new tumor entities and their subtypes and to provide detailed prognostic and pathogenetic information [1]. However, it must be emphasized that molecular testing alone is not sufficient to make the correct diagnosis in head and neck pathology. In fact, on top of the existing histologic morphologic entities, now, molecular biology provides additional information that helps in fine-tuning subtypes and boundaries that previously were not crystal clear. Moreover, the availability of an expanding spectrum of immunohistochemical markers facilitates the rapid identification of useful diagnostic molecular features. As such, it is currently possible for head and neck pathologists to benefit from a molecularly defined classification while still making diagnoses based largely on histopathology and immunohistochemistry.

In recent years, considerable progress in sinonasal tumor taxonomy has taken place with the discovery of tumor type-specific fusion oncogenes generated by chromosomal translocations (such as *DEK::AFF2* and *NUTM1* gene fusions), as well as recognition of inactivated tumor suppressor genes, such as SWI/SNF deficiency (detectable by immunohistochemistry), unique to specific tumor types. This review covers the principal molecular alterations that are important in sinonasal malignant neoplasms from a practical standpoint for diagnosis, prognosis, and prediction of response to treatment. Currently, new therapeutic approaches for sinonasal malignant tumors are urgently needed. For most histological subtypes, surgery is considered the gold standard of treatment when feasible, frequently complemented by adjuvant radiotherapy [2]. Survival has not improved significantly in recent years with this traditional approach, and ongoing efforts focus now on combining existing treatment strategies such as induction chemotherapy with preceding innovative radiation techniques. However, the results remain often disappointing [3]. We aim to contribute to the appreciation of the intersecting roles of molecular testing and more conventional diagnostic modalities in sinonasal tumors.

## Sinonasal epithelial malignant neoplasms

The sinonasal tract, comprising the nasal cavity, the paranasal sinuses, and the anterior skull base, is an anatomic region characterized by a broad spectrum of tumors that exhibit a significant morphological diversity of molecularly defined entities (Table 1). The recent 5th edition of the *WHO Classification of Head and Neck Tumours* includes new entities in which molecular genetics have an important diagnostic role, including HPV-related multiphenotypic sinonasal carcinoma, SWI/SNF-deficient sinonasal carcinoma and adenocarcinoma [1], and a subset of emerging entities including the IDH-mutated malignancies classified in the category of sinonasal undifferentiated carcinoma (SNUC) [1]. Molecular genetics also plays a diagnostic role in the context of hereditary syndromes manifested in the sinonasal tract.

**Table 1:** Molecularly defined sinonasal epithelial malignancies—immunohistochemical features and molecular genetic findings

Tumor type	Immunohistochemistry	Molecular genetic
Respiratory epithelial lesions—carcinomas		
Keratinizing squamous cell carcinoma	CK, p63/p40, CK5/6	*TP53* mutation; *EGFR* mutation; *PTEN*, *CDKN2A*, *KMT2D* alterations
Non-keratinizing squamous cell carcinoma	CK, p63/p40, CK5/6, p16	HPV association (20–62%); type 16; EBV association—rare; subset *DEK::AFF2*
NUT carcinoma	NUT, p63, CD34	*NUTM1::BRD4/BRD3/NSD3/ (ZNF532*, *ZNF592)*
SMARCB1-deficient carcinoma	CK5, p63, CK7, SMARCB1-loss	*SMARCB1* mutation + / − *SMARCA2* mutation
SMARCB1-deficient adenocarcinoma	CK7, p40, glypican 3, SALL4, HepPar-1, CDX2, CK20, PLAP, and AFP; SMARCB1-loss	*SMARCB1* mutation
SMARCA4-deficient carcinoma	CK, CK7, synaptophysin; chromogranin CD56; SMARCA4 loss	*SMARCA4* mutation + / − *SMARCA2* mutation
Sinonasal undifferentiated carcinoma	Diagnosis per exclusionem, a subset IDH1/2	*IDH wild type and IDH1/2* mutations
Teratocarcinosarcoma	According to histological component; SMARCA4 loss, nuclear β-catenin	*SMARCA4* mutation; *CTNNB1* mutation
HPV-associated multiphenotypic sinonasal carcinoma	Biphasic, S100, SOX10, p16	HPV—mainly serovar 33
Sinonasal adenoid cystic carcinoma, including metatypical variant	Biphasic, CK7, S100, SOX10, p63, p40, MYB +	*MYB::NFIB; MYBL1::NFIB*; losses of 1p, 6q, and 15q; mutations in *FGF/IGF/PI3K* and *NOTCH*

Excluded units without diagnostic molecular genetics. Adapted from WHO Classification of Tumours Editorial Board. Head and neck tumours. Lyon (France): International Agency for Research on Cancer; forthcoming. (WHO classification of tumours series, 5th ed.; vol. 9). https://publications.iarc.fr

### Non-keratinizing squamous cell carcinoma including those with DEK::AFF2 gene fusion

Sinonasal non-keratinizing squamous cell carcinoma (NKSCC) comprises a heterogeneous category of neoplasms of different etiological and molecular pathogenesis. It became increasingly evident that NKSCC as a merely descriptive diagnosis is not valid anymore. Notably, a subset of these tumors is driven by transcriptionally active human papilloma virus (HPV), most commonly type 16, in 36–58% of patients with this diagnosis. While routine HPV testing is not recommended in sinonasal NKSCC, it can occasionally be helpful for diagnostic purposes. If HPV testing is carried out, HPV-specific tests such as in-situ hybridization or PCR must be used, as p16 immunohistochemistry has poor specificity in sinonasal tumors [1]. More recently, it has been found that more than half of the NKSCCs that are not associated with HPV reveal a recurrent translocation *DEK::AFF2* [1]*.* These new developments point to the need for adopting a “diagnosis by exclusion” strategy for “NKSCC NOS” after the exclusion of these genetically defined subtypes.

### DEK::AFF2 carcinoma

*DEK::AFF2* carcinoma is currently classified as an emerging entity under the category of non-HPV-associated NKSCC, localized especially in the sinonasal tract; although, a few cases have been reported arising in the middle ear, temporal bone, orbita, and lung [4, 5]. Despite bland-looking morphology, this tumor behaves in an aggressive fashion with a high risk of local recurrence, metastatic nodal dissemination, and distant spread [6].

**Fig. 1:**
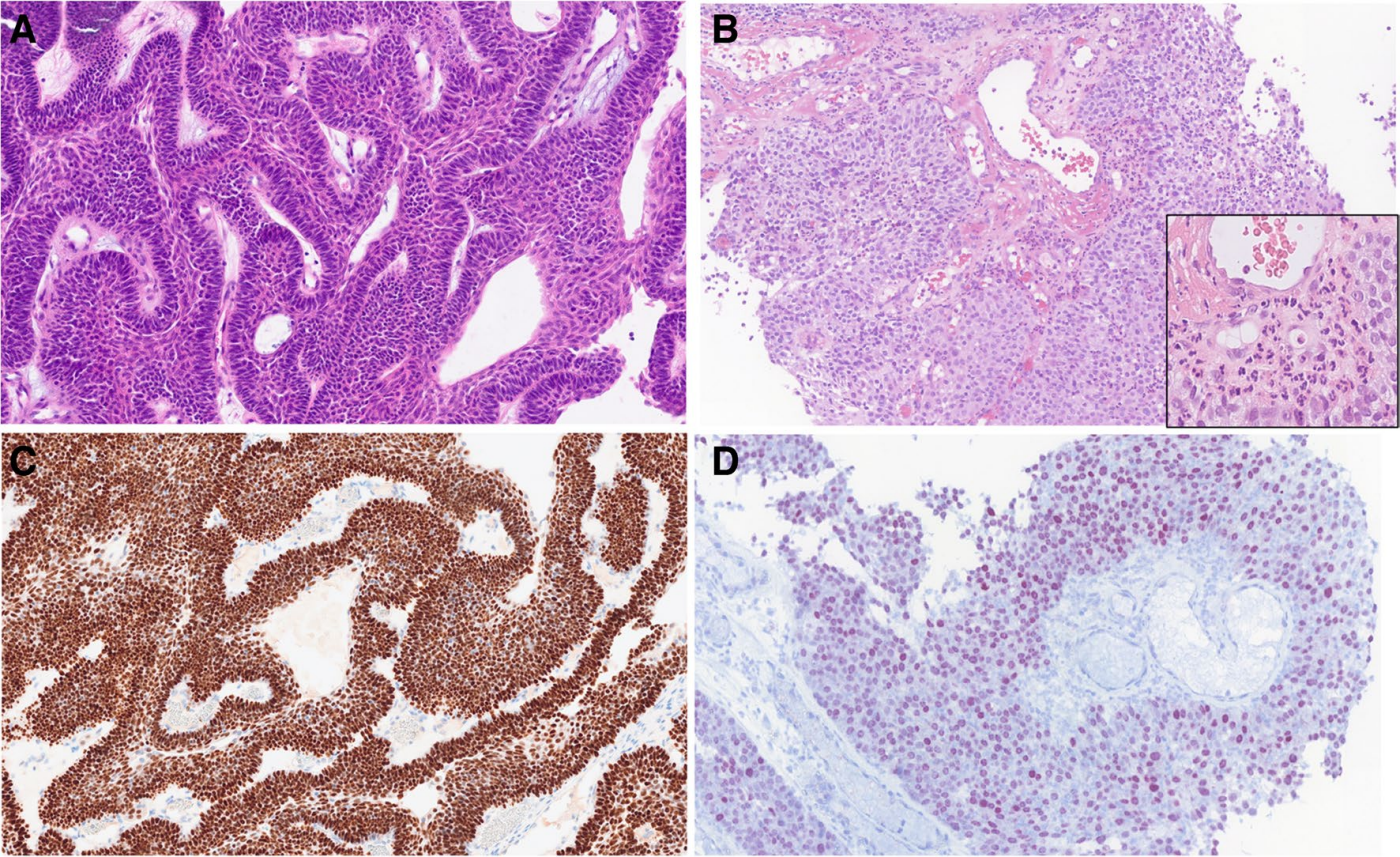
*DEK::AFF2* carcinoma. Nuclear palisading is frequently seen at the periphery of tumor lobules (**A**). Most tumors are densely infiltrated by abundant neutrophils (inset) in both the epithelium and the stroma (**B**). All *DEK::AFF2* carcinomas are diffusely positive for p63 and p40 (**C**) *DEK::AFF2* carcinomas were successfully immunostained for AFF2 protein, and all cases showed positive nuclear expression (**D**)

Histologically, most cases demonstrate a complex exophytic and endophytic growth of basaloid to transitional cells [7, 8]. Where present, the appearance of papillary fronds ranges from delicate to broad [8]. The tumor cells also grow into the underlying stroma forming anastomosing lobules, ribbons, and occasional nests and cords. The invasive pattern tends to be broad-based and pushing, but it may reveal a discohesive pattern of invasion with numerous small irregular nests widely infiltrating into the bone [7]. Nuclear palisading is frequently seen at the periphery of tumor lobules (Fig. 1A). Intraepithelial cell discohesion may result in pseudopapillary formation and stellate reticulum-like appearance in the center of tumor sheets. Typically, tumor cells possess bland-looking, monotonous, round to oval-shaped nuclei with fine to vesicular chromatin, prominent nucleoli, amphophilic to eosinophilic cytoplasm, and indistinct cell borders. The mitotic counts are usually low but a high mitotic index is also seen in a few cases [7, 9]. Tumor necrosis and apoptotic bodies are noted in some cases. Most tumors are densely infiltrated by abundant neutrophils in both the epithelium and the stroma (Fig. 1B) [7–9]. Microabscess formation can occur [9].

All *DEK::AFF2* carcinomas are diffusely positive for p63 and p40 (Fig. 1C) [5]. They are also positive for cytokeratins, including AE1/AE3 and CK5/6. *DEK::AFF2* carcinomas have been successfully immunostained for AFF2 protein, and all cases showed positive nuclear expression (Fig. 1D) [10]. Accordingly, AFF2 immunohistochemistry represents an emerging highly sensitive and specific ancillary marker that distinguishes *DEK::AFF2* carcinoma from other sinonasal tumors with overlapping morphological features, and it may also be useful in decalcified specimens [10]. The original case report on the entity describes a *DEK::AFF2* carcinoma with an excellent response to immune checkpoint inhibitors (ICI)—anti-PD-L1—which is related to *DEK::AFF2* neoantigen-specific T-cell response during tumor regression [4].

### Sinonasal NUT Carcinoma

**Fig. 2:**
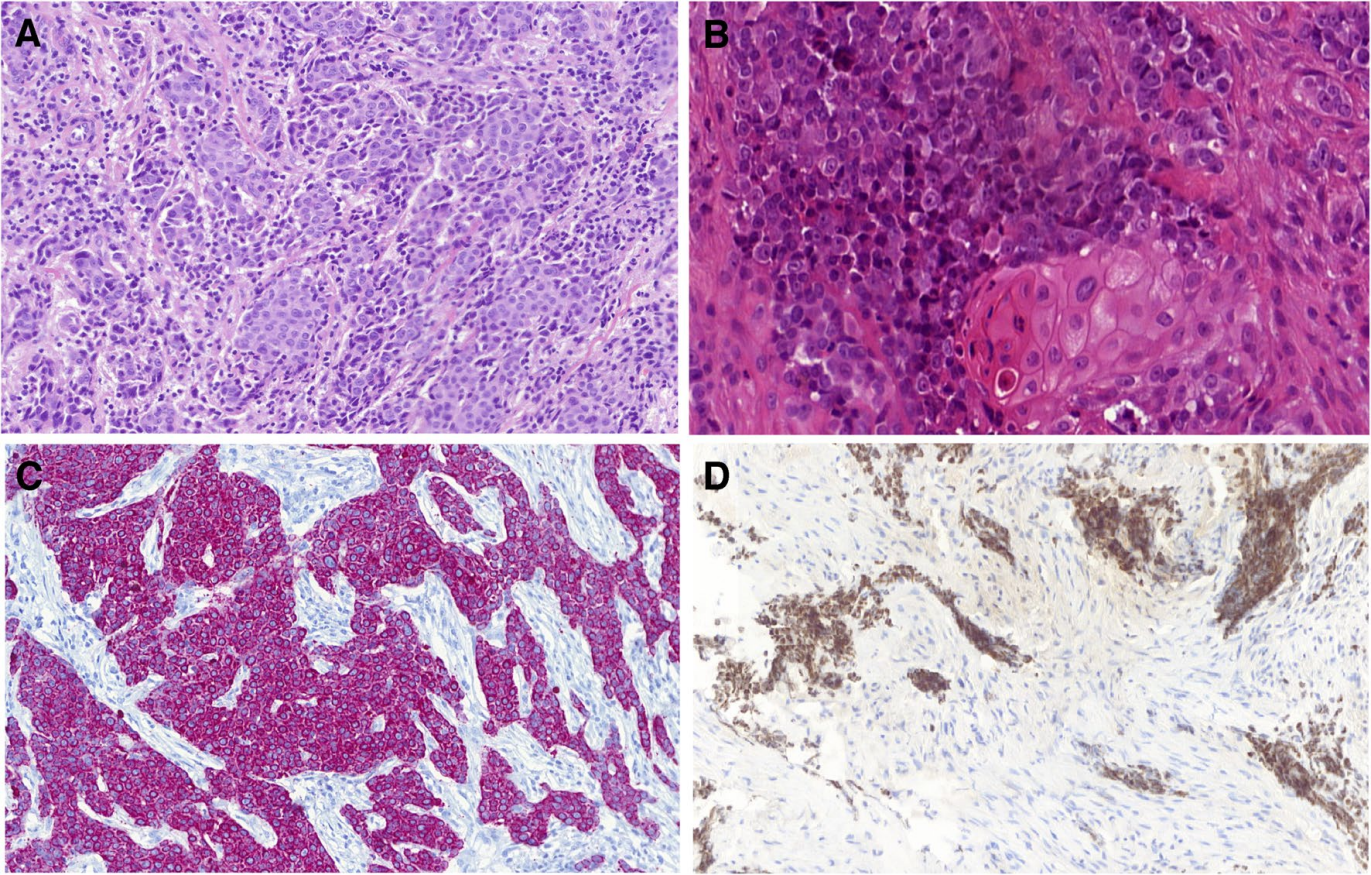
NUT carcinoma. Nuclei are relatively uniform and monotonous (**A**). In some NUT carcinomas, abrupt keratinization is seen; undifferentiated tumor cells are present immediately next to highly differentiated keratin pearls (**B**). The tumor cells are strongly positive for cytokeratin CK5/6 (**C**). The monoclonal NUT antibody is highly specific for NUT carcinoma (**D**)

NUT Carcinoma is a highly aggressive, mostly lethal malignancy with monotonous poorly differentiated morphology. NUT carcinoma (formerly termed NUT midline carcinoma) has a predilection for mediastinum (approximately 50% of cases) and head and neck, particularly the sinonasal tract but also elsewhere, for example, the larynx [11]. Histologically, NUT carcinoma is a very poorly differentiated malignancy that grows as nests and sheets of tumor cells. NUT carcinoma is a highly infiltrative and cytologically high-grade malignancy with numerous mitotic figures and frequent tumor necrosis. A clue to diagnosis is the fact that despite the tumor being clearly high-grade, tumor nuclei lack significant pleomorphism that is typically seen in high-grade carcinomas. In contrast, the nuclei are relatively uniform and monotonous (Fig. 2A). In some NUT carcinomas, overt squamous differentiation is seen in the form of either ‘‘abrupt” keratinization, i.e., undifferentiated tumor cells are often seen immediately next to highly differentiated keratin pearls or as ‘‘abrupt’’ squamoid cells aggregates with copious clear cytoplasm within the undifferentiated basaloid cell aggregates (Fig. 2B). This overt squamous differentiation is seen in no more than 43% of cases [12].

NUT carcinoma is considered to be a part of the spectrum of squamous cell carcinomas, which is supported by positivity for cytokeratin, CK5/6 in particular (Fig. 2C), and p63, while p40 is less reliably positive. The monoclonal NUT antibody is highly specific for NUT carcinoma (Fig. 2D) [13]. NUTM1-rearranged tumors such as skin adnexal poroid neoplasms and *CIC::NUTM1* sarcoma are positive for NUT antibody by immunohistochemistry, but these tumors are almost never sinonasal [14, 15]. Limited focal expression of the wildtype NUT protein can be seen rarely in some conventional squamous cell carcinomas, but the staining is weak and focal, being present in < 20% of neoplastic cells. The typical punctate pattern of positivity is limited to NUT carcinoma, and it is seen in > 70% of cells and is often uniformly positive in all tumor cells.

NUT carcinoma is genetically characterized by a rearrangement of the *NUTM1* gene (Nuclear Protein in Testis) on chromosome 15q14 [16]. The *NUT* gene is physiologically expressed in mature spermatogonia. The NUT-signaling molecule binds to histone acetyltransferase (HAT) p300 and activates histone acetylation. The most common fusion partners of NUT are genes involved in transcription and chromosome regulation belonging to the BET family (*BRD2*, *BRD3*, *BRD4*, and *BRDT*) [17]. In 75% of cases, the fusion partner of *NUTM1* is the *BRD4* gene (in 19p13.1), followed by *BRD3* (in 9q34.2) in 15% of cases [18]. The NUT::BRD3/4 fusion oncoprotein functions by blocking cell differentiation and promoting uncontrolled cell growth [19]. In a subset of cases, *NUT* is fused with non-BRD genes. In 6% of the cases, the fusion involves the *NSD3* gene (in 8p11.23), which codes for an oncoprotein required for differentiation and regulation of cell proliferation [20]. In 2% of the cases, genes for zinc finger-containing proteins, (*ZNF532* on 18q21.32 or *ZNF592* on 15q25.3) are involved in *NUT* fusion [21].

Until recently, there was no known effective treatment for NUT carcinoma which has a median survival of 6.7 months, explaining the name “ticket to heaven” for this aggressive entity [22]. Surgery and radiotherapy form the gold standard, and they may prolong progression-free survival and overall survival (OS); recently, somewhat better results were seen using an induction chemotherapy strategy [23]. In a recent study of 12 patients with sinonasal NUT carcinoma treated at MD Anderson Cancer Center, the median OS was 14.6 months. Patients with maxillary sinus tumors were 91% more likely to survive (hazard ratio [HR], 0.094; 95% confidence interval [CI], 0.011–0.78, *p* = 0.011). Patients with higher-stage disease stage had worse OS (stage IVb-c no 2-year survivors, vs. stage III-Iva 60% 2-year survival, *p* = 0.05). All three patients who were alive with no evidence of disease received induction chemotherapy [24]. The first targeted drugs for NUT carcinoma were histone deacetylase inhibitors (HDACi) (vorinostat) and BET inhibitors (BETi), which inhibit tumor cell growth and induce cell differentiation [25]. BETi (JQ1) molecules mimic acetylated histones and competitively inhibit the tethering of BRD3/4 to acetylated chromatin. In addition, BETi directly targets the NUT fusion protein [19]. Whether such targeting in NUT carcinoma results in a clinical response has not been investigated yet. Furthermore, no BETi has been granted FDA approval to date [26].

## SWI/SNF complex deficient sinonasal carcinoma and other malignancies

The chromatin remodeling Switch/Sucrose non-fermentable complex (SWI/SNF) is a pleomorphic complex of over 20 tumor suppressors that communicate with transcription factors at the promotor site, mobilize nucleosomes, and modulate chromatin structure [27]. These genes are involved in cell differentiation and proliferation. There are four different subtypes of SWI/SNF complex deficient sinonasal/base of skull malignancies, including SMARCB1-deficient sinonasal carcinoma, SMARCB1-deficient sinonasal adenocarcinoma, SMARCA4-deficient sinonasal carcinoma, and a subset of SMARCA4-deficient teratocarcinosarcomas. Poorly differentiated chordomas are also SMARCB1-deficient, and they may rarely involve the sinonasal tract by extension from the skull base.

### SMARCB1-deficient sinonasal carcinoma

SMARCB1 deficiency in a subset of poorly or undifferentiated sinonasal carcinoma was first recognized in 2014 by 2 independent groups [28, 29] followed by a few additional case series, the largest multi-institutional series comprised 39 patients [30]. SMARCB1-deficient sinonasal carcinoma is defined by a lack of features of any other defined sinonasal carcinoma type, complete loss of SMARCB1 expression, and absence of morphologic squamous or glandular differentiation. Most cases show basaloid cell morphology but squamous features and keratinization are absent. Around 30% of cases, however, show eosinophilic cells with rhabdoid and/or plasmacytoid features. The tumor cells usually display large solid nests and sheets. Immunohistochemically, the neoplastic cells of SMARCB1-deficient sinonasal carcinoma are uniformly pan-cytokeratin positive, focally positive for p63/p40, and negative for NUT and p16 [31].

### SMARCB1-deficient sinonasal adenocarcinoma

**Fig. 3:**
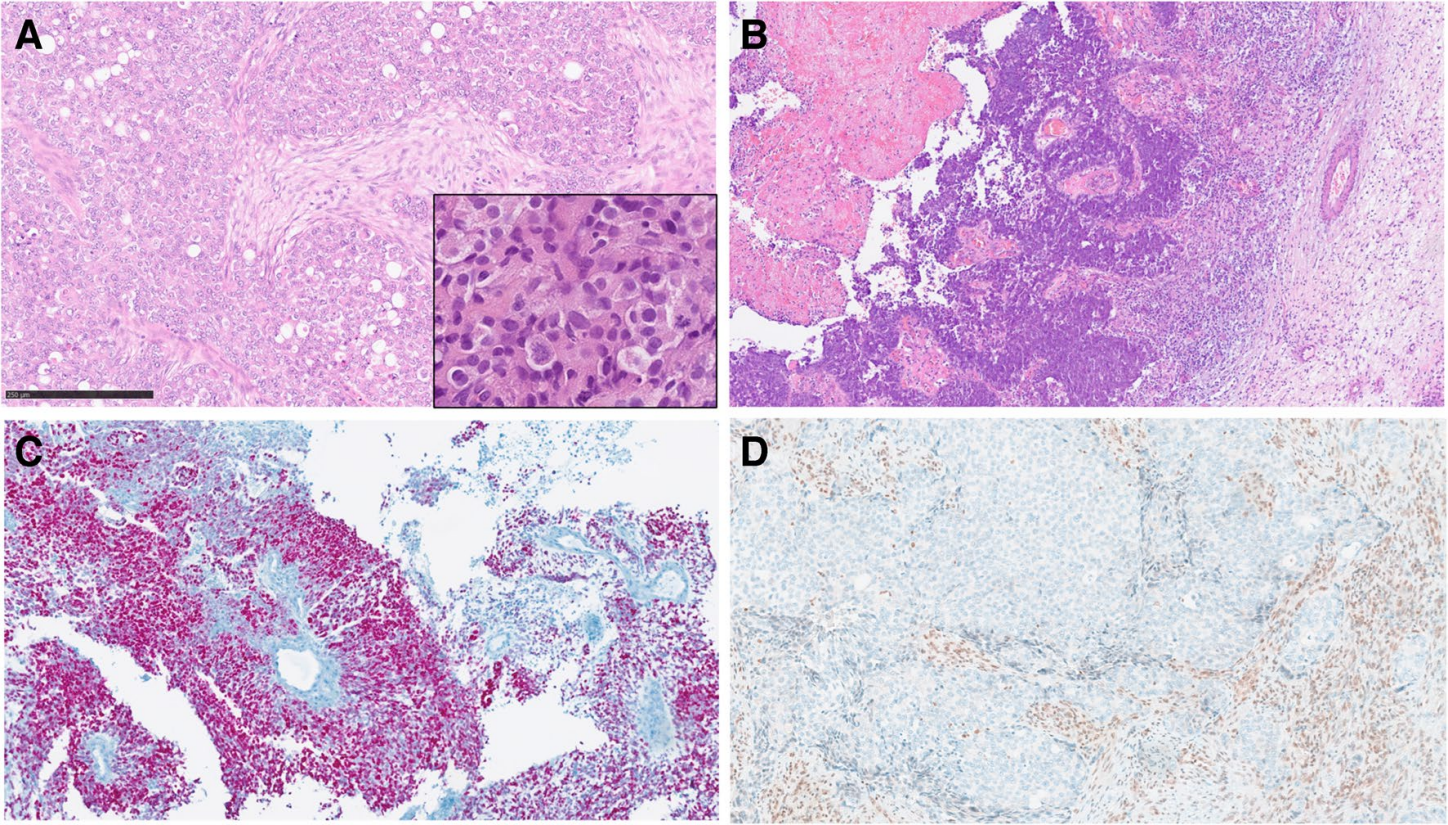
SMARCB1 deficient adenocarcinoma of the nasal cavity. Tumor cells are large (inset) with eosinophilic, oncocytoid, plasmacytoid, and/or rhabdoid appearance (**A**). Areas with yolk sac tumor-like differentiation including Schiller–Duval body-like structures are found (**B**). Immunohistochemical staining for yolk sac tumor marker SALL4 is often positive (**C**). Tumor cells are devoid of INI1 immunostaining (**D**)

This is a rare SWI/SNF-deficient malignancy defined by the presence of unequivocal glandular differentiation and/or by the presence of other features of adenocarcinoma [32]. Tumor histomorphology is predominantly solid, with trabecular and alveolar growth patterns. The tumor cells are large with eosinophilic, oncocytoid, plasmacytoid, and/or rhabdoid appearance (Fig. 3A). SMARCB1-deficient sinonasal adenocarcinoma demonstrates varying proportions of glandular formations, including alveolar/acinar structures with abortive microglandular differentiation, trabecular, and solid/cribriform/insular patterns. Areas with yolk sac tumor-like differentiation including Schiller–Duval body-like structures are often found (Fig. 3B). Immunohistochemical markers for yolk sac tumor (SALL4 or glypican-3) are often seen (Fig. 3C), corresponding to their yolk sac tumor-like histologies. SMARCB1-deficient sinonasal adenocarcinomas lack the basaloid morphology seen in 60–70% of SMARCB1-deficient sinonasal carcinomas and instead display either clear-cut gland formation, cribriform patterns, or mucin production. Some of them are reminiscent of high-grade non-intestinal type adenocarcinoma while others may closely mimic myoepithelial carcinomas. The yolk sac tumor-like pattern is limited to the adenocarcinoma subgroup. While focal p63 and/ or CK5/6 immunopositivity can be seen, they lack the diffuse uniform pattern of squamous cell type, and yolk sac markers are frequently expressed. However, tumors with transitional features between the two types are seen indicating a morphological spectrum.

### SMARCA4-deficient sinonasal carcinoma

Since the first detailed description by Agaimy and Weichert [33], no more than 22 cases of SMARCA4-deficient sinonasal carcinoma have been reported in the literature [31]. These highly aggressive tumors represented 4% of all undifferentiated and poorly differentiated sinonasal carcinomas and 9% of tumors that have been previously classified as SNUC. This figure relates to archival diagnoses and not to the current classification system [31]. The majority of SMARCA4-deficient carcinomas develop in the nasal cavity, and they involve multiple sinonasal sites in a subset of cases. Because of their large monotonous cell morphology and their frequent neuroendocrine features, they have been frequently initially misclassified as neuroendocrine carcinomas. Histologically, SMARCA4-deficient carcinomas are undifferentiated, hence closely akin to SNUC. They are arranged in sheets of large anaplastic epithelioid cells disposed into irregularly communicating nests and lobules or trabeculae within a sparse to prominent reactive edematous or desmoplastic intervening stroma.

### SMARCA4-deficient teratocarcinosarcomas

This rare multi-phenotypic (trilineage) and highly aggressive site-specific sinonasal malignancy is still defined by morphology in the current World Health Organization classification, but it merits mentioning here due to overlapping molecular features with SWI/SNF-deficient sinonasal carcinomas. Teratocarcinosarcoma (TCS) is defined by a triphasic growth of teratoma-like (embryonal epithelium of diverse types, neuroectodermal differentiation, and primitive neuroepithelium), carcinoma-like (either malignant differentiated epithelial elements or keratin-positive poorly differentiated proliferations), and sarcomatous stromal/mesenchymal elements with (mainly rhabdomyoblastic and rare osteochondroblastic) or without heterologous mesenchymal elements [34]. Recurrent loss of SMARCA4 expression in more than 80% of TCS cases was recently described [34]**.** However, in contrast to SWI/SNF-deficient sinonasal carcinoma types described above, where the SWI/SNF defect is definitional, sinonasal TCS is still defined morphologically based on the histological criteria set stated above [34].

SWI/SNF complex deficient sinonasal tumors are usually poorly differentiated or undifferentiated malignancies with a highly aggressive clinical course and poor patient outcomes. The mortality of SMARCA4-deficient sinonasal carcinomas is higher than in other tumors of this family [30, 35].

Differential diagnosis is challenging, and it is mainly defined by the histological pattern in any individual case. Non-keratinizing SCC (sporadic or HPV-related) and basaloid variants of many other entities such as NUT carcinoma, adamantinoma-like Ewing sarcoma, and neuroendocrine carcinomas must be considered. Large cell SWI/SNF-complex-deficient tumors need to be distinguished from SNUC, rare variants of NUT carcinoma, melanoma, dedifferentiated chordomas, aggressive anaplastic lymphomas, and metastases. The availability of immunohistochemical antibodies to SWI/SNF proteins represents an effective tool for the identification of these neoplasms in the appropriate clinicopathological and morphological context. SWI/SNF-complex-deficient malignancies can be determined by immunohistochemical staining with INI1 antibody for SMARCB1-deficient and BRG1 antibody for SMARCA4-deficient carcinomas. These antibodies are sensitive diagnostic tools (Fig. 3D) [31].

Regarding therapy, available data on the significance of the distinction between SMARCB1-deficient sinonasal carcinoma and adenocarcinoma as well as their long-term follow-up is limited. Currently, this distinction should enable reliable assessment of any therapeutic or prognostic differences between the two. Patients are often treated by radical surgery and adjuvant radiotherapy or radiochemotherapy. Despite this aggressive treatment, a recent multicenter case series revealed that only one of three patients was alive and free of tumors at a median follow-up of slightly more than 2 years. [25] The alternative therapeutic option rests on local control of the disease with polychemotherapy and radiotherapy [30, 35]. The largest single-institution study of SMARCB1-deficient sinonasal carcinoma to date reported the outcomes of 19 consecutive patients with SMARCB1 (INI-1)-deficient sinonasal carcinoma treated at the MD Anderson Cancer Center. The median overall survival (OS) and disease-free survival (DFS) were 31.8 and 9.9 months, respectively. Patients with nasal cavity or maxillary sinus tumors had 84% better disease-specific survival (DSS) (hazard ratio [HR], 0.136; 95% confidence interval [CI], 0.028–0.66; *p* = 0.005) and 71% better DFS (HR, 0.29; 95% CI, 0.097–0.84; *p* = 0.041) than patients with other sinonasal sites. Patients who received induction chemotherapy were 76% less likely to die of disease (DSS HR, 0.241; 95% CI, 0.058–1.00; *p* = 0.047) [36].

Recent findings suggest a promising role for immunomodulators and immune checkpoint inhibitors as potential drugs for patients with SWI/SNF-related malignancies [27, 37]. EZH2 inhibitor, a histone methyltransferase, activates the methylation of histone H3 at lysin 27 (H3K27me3), resulting in the epigenetic silencing of cell fate-associated genes. Tumor cells with loss of *SMARCB1* demonstrate a constitutive EZH2 activation and oncogenic activation [27, 37]. EZH2 inhibitors may modulate tumor immunogenicity and anti-tumor immune responses [38].

### Sinonasal undifferentiated carcinoma

Sinonasal undifferentiated carcinoma (SNUC) is a high-grade epithelial neoplasm without any lines of differentiation, and the diagnosis is made only after the exclusion of other sinonasal and non-sinonasal malignancies. In the previous WHO classifications, many undifferentiated epithelial neoplasms were included under this term, until advances in molecular pathology allowed for their identification as separate entities. Recently, isocitrate dehydrogenase 1 or 2 (*IDH1/2*) mutations were identified in a subset of SNUC [39, 40], including three main hotspot mutations of *IDH1 R132*, *IDH2 R140*, and *IDH2 R172* [41]. Monoclonal or multi-specific antibodies for the detection of IDH1/2 mutations represent an alternative cheaper than molecular genetic testing, while immunohistochemistry using the present antibodies lacks the ability to detect the full spectrum of IDH1/2 mutations [42]. We recommend using both immunohistochemistry and molecular analysis. The *IDH* proteins participate in the Krebs cycle converting isocitrate to α-ketoglutarate. *IDH* mutations produce an oncometabolite, D-2-hydroxyglutarate (2-HG), that induces DNA hypermethylation.

SNUCs are aggressive neoplasms with a silent clinical course until they are diagnosed at an advanced stage, followed by a rapid progression and poor outcome. The standard therapeutical approach used to be a combination of surgery, chemotherapy, and radiation [43], but the optimal sequence of these treatments has been long debated. Recently, a landmark study on 95 previously untreated patients reported improved outcomes using a curative intent strategy of induction chemotherapy (IC). In SNUC patients who received this treatment, the 5-year DSS was 59% (95% CI, 53 to 66%). The response to IC determined whether concurrent chemoradiation was continued. Responders to IC with complete response (CR) or partial response (PR) showed an 81% 5-year DSS. Non-responders received surgery and postoperative radiotherapy and showed a 54% 5-year DSS [43, 44].

However, in patients who did not experience even a partial response to IC with concurrent chemoradiotherapy (CRT), the 5-year DSS was 0%. In patients without even partial response to IC and those who were treated with surgery plus radiotherapy or CRT, the 5-year DSS was 39% (adjusted hazard ratio of 5.68 [95% CI, 2.89 to 9.36]). The authors concluded that in patients who achieve a favorable response to IC, definitive CRT results in improved survival compared with those who undergo definitive surgery. In patients who do not achieve a favorable response to IC, surgery when feasible seems to provide a better chance of disease control and improved survival [44].

Tumors with *IDH2* mutations show a better outcome than other SNUCs [43]. The presence of *IDH* mutations provides alternative therapeutic options including selective small molecule inhibitors (e.g. Enasidenib for *IDH2* mutations and Ivosidenib for *IDH1* mutations) which inhibit DNA hypermethylation and lead to delayed cancer cell growth and induction of cell differentiation [45].

### HPV-associated multiphenotypic sinonasal carcinoma

HPV-associated multiphenotypic sinonasal carcinoma (HMSC) is an epithelial malignancy localized almost exclusively in the sinonasal tract and harboring high-risk HPV [46]. The most common serovariety is type 33, followed by other also rare serotypes such as 52, 56, and others. HMSC immunohistochemistry is positive for p16 and high-risk HPV testing using direct assays such as RNA in situ hybridization is also positive. A negative p16 result helps to exclude this tumor type, but positive p16 can be non-specific and cannot be used as an HPV surrogate in this tumor. HMSC is histologically very pleomorphic and may mimic different salivary and non-salivary tumor types. HMSCs are characterized by a dual population of ductal and myoepithelial cells, mainly solid growth patterns, areas of necrosis, as well as overlying involvement of the surface epithelium similar to high-grade dysplasia. In places, HMSC may histologically resemble adenoid cystic carcinoma (AdCC), which is an important differential diagnosis, as the prognosis of HMSC is usually good unlike true AdCC [47]. The histological appearance of HMSC is usually associated with high-grade cytomorphology and a destructive growth and propensity for local recurrence. Despite the aggressive appearance, HMSC has low metastatic potential and little tendency to lethal behavior [48].

## Sinonasal adenoid cystic carcinoma

**Fig. 4:**
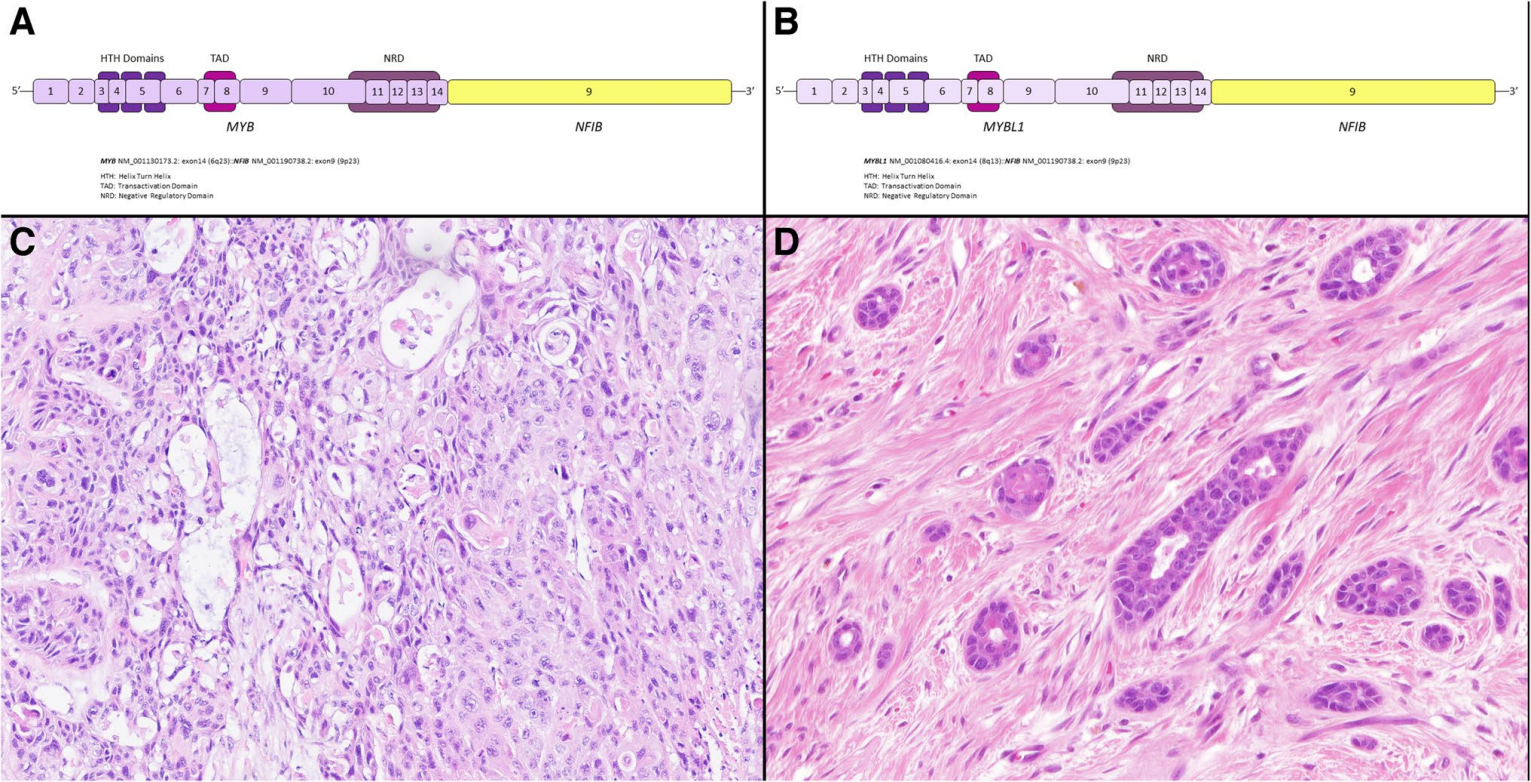
Sinonasal adenoid cystic carcinoma (AdCC). The fusions joining of *MYB* gene exon 14 with *NFIB* gene exon 9 (**A**) and *MYBL1* gene exon 14 with *NFIB* gene exon 9 (**B**) are illustrated. Protein domains are depicted. Metatypical AdCC with prevailing solid pattern and focal squamous differentiation (**C**). Another unusual histological pattern of metatypical AdCC is a striking tubular hypereosinophilia (**D**)

Adenoid cystic carcinoma (AdCC) is an invasive malignancy composed of epithelial and myoepithelial neoplastic cells arranged in tubular, cribriform, and solid patterns associated with an eosinophilic extracellular matrix and reduplicated basement membrane materials, and often with gene fusions involving the *MYB*, *MYBL1*, and *NFIB* genes. The genomic hallmarks of AdCC are t(6;9) or t(8;9) translocations, resulting in *MYB::NFIB* and *MYBL1::NFIB* fusions, respectively [49, 50] (Fig. 4A and B). The former alteration is found in > 80% of the cases and the latter in approximately 5% [50]. *MYB/MYBL1* activation due to gene fusion or other mechanisms is a key event in the pathogenesis of AdCC [49]. Losses of 1p, 6q, and 15q are associated with high-grade tumors, while loss of 14q is seen exclusively in low-grade tumors [51, 52].

Metatypical AdCC has been recently recognized as a morphological variant of AdCC with a predilection to the sinonasal mucosa [53, 54]. Metatypical AdCCs have been noted for unusual patterns, including squamous differentiation and macrocystic growth (Fig. 4C) [53]. Another unusual histological pattern of metatypical AdCC is a striking tubular hypereosinophilia and luminal cell prominence which are in contrast to the vast majority of AdCC that are basaloid with myoepithelial cell predominance (Fig. 4D) [54].

Next-generation sequencing of AdCC has identified mutations with mostly low levels of recurrence in genes of the *FGF/IGF/PI3K*, chromatin remodeling, and *NOTCH* signaling pathways [55, 56]. Traditional therapeutic agents for patients with advanced, recurrent, or metastatic AdCC have demonstrated poor efficacy in prolonging survival. Therefore, significant efforts have been undertaken to develop targeted therapies that could improve the outcome. In particular, the presence of *NOTCH* alterations in AdCC was shown to be associated with poor survival. Patients with these aberrations might potentially benefit from anti-NOTCH drugs, such as Brontictuzumab [57]. In the same line, AL101 (osugacestat) has been shown to have potent antitumor effects in in vitro and in vivo models (AdCC cell lines, organoids, and patient-derived xenograft models) of AdCC with activating *NOTCH1* mutations [58].

In a very recent study, high expression of B7-H4 (VTCN1), a member of the B7 family, was associated with a poorer prognosis in AdCC, regardless of clinical stage and histologic subtype. B7-H4 expression was particularly high in solid ACC and was an independent prognostic marker in this disease [59]. Another recent study from MD Anderson Cancer Center demonstrated that the cooccurrence of multiple actionable protein/pathway alterations in various subtypes of AdCC indicates unique therapeutic vulnerabilities and opportunities for optimal combination therapy for this understudied and heterogeneous disease [60].

## Soft tissue neoplasms with sinonasal predilection

### Molecular alterations with differential diagnostic significance

The head and neck region can be a host to a wide range of soft tissue neoplasms, but a discussion of all these entities is beyond the scope of this review. Thus, we have included only soft tissue malignancies that occur frequently or are unique to the sinonasal tract with diagnostically useful molecular alterations. The discussed entities are listed in Table 2.

**Table 2:** Mesenchymal tumors of head and neck region—typical location, important immunohistochemical features and diagnostically useful molecular genetic data

Tumor type	Typical location(s)	Immunohistochemistry	Molecular genetics
Rhabdomyosarcomas (RMS)			
Alveolar RMS	Oral cavity, sinonasal area	PAX3/7-FOXO1, MYOD1, desmin, myogenin	*PAX3::FOXO1* (or *PAX7::FOXO1*)
VGLL2/3, NCOA1, CITED1 rearranged RMS	Rarely	MYOD1, desmin, myogenin	*VGLL2/3*, *SRF, TEAD1*, *NCOA2*, and *CITED2* rearrangements
MyoD1 mutant RMS	Rarely	MYOD1, desmin, myogenin	*MYOD1 p.Leu122Arg* gene mutation
TFCP2 rearranged RMS	Mandible, maxilla	MYOD1, desmin, myogenin, CK, ALK (50%)	*EWSR1/FUS::TFCP2*, *ALK* alterations
Malignant peripheral nerve sheath tumor	Soft tissue of the neck	S100 (50%)/SOX10 (70%); H3K27me3 loss	*NF1* inactivation, *SUZ12*, or *EED* mutations
Tumors of uncertain differentiation			
Phosphaturic mesenchymal tumor	Sinonasal tract, mandible, maxilla	ERG, SATB2, CD56, SSTR2A	*FN1::FGFR1*, *FN1::FGF1*
Extraskeletal myxoid chondrosarcoma	Orbit, intracranial, and sinonasal area	Not diagnostically useful	*NR4A3* rearrangements *(EWSR1*, *TAF15*, *TCF12*, *TFG*, *FUS*, *HSPA8)*
Synovial sarcoma	Head and neck	SS18-SSX, SSX	*SS18::SSX1/2/4*
*GLI1*-altered soft tissue tumor	Tongue, submandibular gland, soft tissues of the neck	GLI1, variable S100, MDM2, CDK4, STAT6 SMA and CK	*GLI1* rearrangement or *GLI1* amplification
Undifferentiated small round cell sarcomas of the bone and soft tissue			
Ewing sarcoma (ES)	Skull and facial bones, soft tissues, mandible	CD99 NKX2.2	*EWSR1/FUS:: FLI1/ERG/ETV1/ETV4/FEV*
Adamantinoma-like ES	Salivary glands, thyroid gland, and sinonasal area	CK, p40, p63, CD99, NKX2.2	*EWSR1::FLI1*
Others			
Biphenotypic sinonasal sarcoma	Sinonasal	SMA, S100, variably desmin, MyoD1	*PAX3* gene rearrangements *(NCOA1*, *NCOA2*, *FOXO1*, *FOXO6*, *WWTR1*, and others)
Ectomesenchymal chondromyxoid tumor	Tongue	GFAP, variably S100, desmin, SMA, CK, EMA	*RREB1::MRTFB* (formerly called *MKL2*) fusions
*NTRK*-rearranged spindle cell neoplasms	Broad distribution including craniofacial bones	Variably S100, CD34, CD30	*NTRK1/2/3*, *BRAF*, *RAF1*, *RET*, *MET*, and other kinase fusions, rarely *BRAF* or *EGFR* mutations
*EWSR1/FUS::POU2AF3* sarcomas	Sinonasal, rarely craniofacial bones	Variably CK, GFAP, S100 protein, neuroendocrine markers, SATB2	*EWSR1/FUS::POU2AF3* (formerly called *COLCA2*) fusions
Chordoma	Sinonasal	Brachyury (TBXT), CK, EMA; + / − S100, CEA, GFAP	*TBXT* mutations *PBRM1/SETD2* alterations, *CDKN2A* deletion; *SMARCB1 deletion

Adapted from the following references: (1) WHO Classification of Tumours Editorial Board. Head and neck tumours. Lyon (France): International Agency for Research on Cancer; forthcoming. (WHO classification of tumours series, 5th ed.; vol. 9). https://publications.iarc.fr. (2) WHO Classification of Tumours Editorial Board. Soft tissue and bone tumours. Lyon (France): International Agency for Research on Cancer; 2020. (WHO classification of tumours series, 5th ed.; vol. 3). https://publications.iarc.fr/588

*SMARCB1 homozygous deletion is present in poorly differentiated chordomas

### Biphenotypic sinonasal sarcoma

**Fig. 5:**
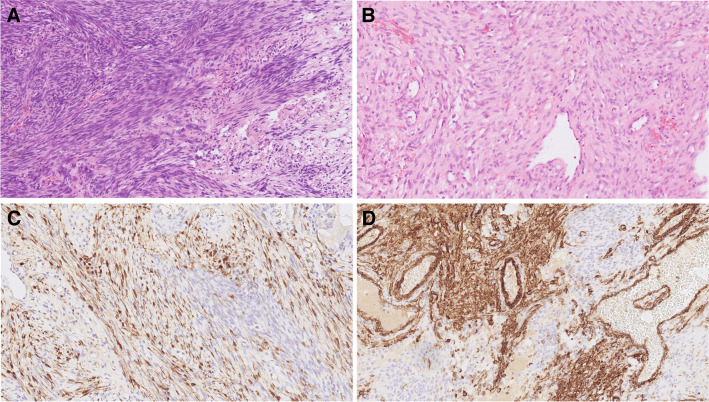
Biphenotypic sinonasal sarcoma. Spindle cell proliferation with fascicular architecture of variable density, some areas are highly cellular (**A**). Hypocelular area with staghorn-like vessels resembling solitary fibrous tumor (**B**). The biphenotypic pattern is highlighted by S100 protein expression (**C**) and smooth muscle actin positivity (**D**)

Biphenotypic sinonasal sarcoma (BSS) is a low-grade spindle cell mesenchymal neoplasm with neurogenic and myogenic differentiation localized exclusively in the sinonasal region. BSS is molecularly defined by the rearrangement of the *PAX3* gene, which is involved in neurogenic, melanocytic, and skeletal muscle differentiation (Fig. 5 A–D) [61]. The most common gene fusion is *PAX3::MAML3* in more than half of the cases, with alternative fusion partners including *NCOA1*, *NCOA2*, *FOXO1*, *FOXO6*, and *WWTR1* [62–64].

BSS is usually a low-grade tumor with local recurrence reported in a third up to a half of the cases, and it may recur many years after diagnosis [65]. Three cases of BSS with high-grade transformation have been published [66–68], one of which developed into rhabdomyosarcoma (RMS). Molecular testing of BSS and RMS showed similar gene fusions including *PAX3::FOXO1* and *PAX3::NCOA1.* Potentially, BSS and RMS form a continuum of lesions [62].

### Ectomesenchymal chondromyxoid tumor

Ectomesenchymal chondromyxoid tumor (ECT) (aka *RREB1::MRTFB-*rearranged neoplasm) is a tumor of uncertain malignant potential located predominantly in the tongue and arising exceedingly rarely in extraglossal locations [69, 70]. The immunoprofile is non-specific and therefore molecular genetic testing is the preferable method when diagnosing ECT. This tumor is characterized in most cases by a *RREB1::MRTFB* fusion, while there is *EWSR1* gene rearrangement in a smaller subset of cases [71, 72]. ECTs are genetically and histologically linked to soft tissue myoepithelial tumors [71] and may be mistaken for other mesenchymal tumors with dominant spindle cell morphology [73]. ECT usually follows a benign course with no metastasis. Surgery is curative in most cases, even though local recurrences may occur [69, 70]. Recently, neoplasms carrying the *RREB1::MRTFB* fusion and showing a variable morphological and immunophenotypic overlap with glossal ECT have been reported from diverse head and neck as well as from non-head and neck sites. Notably, 5 of 10 reported cases, reviewed in [73], involved the parapharyngeal space and the mandible/or sinonasal tract. One reported sinonasal tumor carrying the *RREB1::MRTFB* fusion was more similar to sinonasal chondromesenchymal hamartoma but lacked *DICER1* alterations known to characterize the majority of sinonasal chondromesenchymal hamartomas [73].

### GLI1-altered soft tissue tumors

GLI1-altered soft tissue neoplasms are recently described mesenchymal tumors of uncertain histogenesis, characterized by epithelioid or glomoid (and less frequently focally spindled) morphology and non-specific immunoprofile, presenting in the head and neck in 40% of cases. In two-thirds of the cases, these tumors harbor *GLI1* fusions including *ACTB::GLI1*, *PTCH1::GLI1*, *MALAT1::GLI1*, and *DERA::GLI1*, while the rest harbor *GLI1* amplification [1, 74, 75]. While the various fusion partners to GLI1 are best determined by targeted RNA-sequencing, the GLI1 amplification can also be detected by FISH. The possibility of co-amplification of the neighboring genes (particularly *CDK4*, *MDM2*, *STAT6*, and *DDIT3)* on chromosome 12 and detected by FISH has to be taken into account [76]. In addition, GLI1 immunostaining shows high specificity and good sensitivity for GLI1-rearranged mesenchymal tumors [77]. The combination of GLI1 IHC and p16 stain is superior in detecting GLI1 amplified neoplasms [78].

The biologic behavior of GLI1-altered soft tissue neoplasms varies from completely indolent to potentially aggressive metastasizing neoplasms. Local recurrence or distant spread may appear in approximately 20% of the cases [75, 79]. Potential targeted therapeutic options in *GLI1*-altered neoplasms include sonic hedgehog signaling pathway inhibitors [80].

### Sinonasal rhabdomyosarcomas

Rhabdomyosarcomas (RMS) are clinically, prognostically, and biologically heterogeneous groups of tumors categorized morphologically into four main subtypes [81]. However, molecular-genetic findings delineate six distinct subtypes; embryonal RMS with unknown driver mutations/fusions, alveolar RMS with *FOXO1* fusions, *MYOD1-*mutated RMS with *MYOD1* activating mutations, *VGLL2/VGLL3/NCOA2*-rearranged RMS, pleomorphic RMS with a complex genetic background, and finally, *TFCP2*-rearranged RMS with *EWSR1* or *FUS* fusion partners [82]. Spindle cell/sclerosing RMS are usually positive (at least focally) for cytokeratins and the myogenic markers, mainly MyoD1, myogenin, desmin, or PAX7. The combination of positivity for pankeratin, ALK, and desmin is highly suggestive of *TFCP2-*fused RMS.

*VGLL2/VGLL3/NCOA2*-rearranged RMS belong to a category of spindle cell/sclerosing RMS, primarily affecting newborns or infants and exhibiting head and neck predilection. The characteristic molecular genetic events involve *VGLL2::CITED3*, *VGLL2::NCOA2*, *TEAD1::NCOA2*, or *SRF::NCOA2* gene fusions [83, 84]. Recently, six cases with novel *VGLL3* rearrangements with *TCF12*, *EP300*, and *PPARGC1A* as fusion partners have been described [85]. Clinically, these tumors have a favorable prognosis. Only four cases with metastases have been reported. Upon relapse, three of them displayed high-grade morphology with mutations in genes encoding cell cycle proteins (particularly *TP53*, *CDKN2A/B*, and *FGFR4*) [86]. Complete surgical resection is usually curative, while RMS-type chemotherapy is recommended for unresectable cases because of the potential for high-grade transformation.

*TFCP2*-rearranged RMS (*TFCP2*-RMS) is a spindle cell/sclerosing and very aggressive mesenchymal tumor with rhabdomyoblastic differentiation, characterized by *EWSR1/FUS::TFCP2* rearrangements [87]. In a subset of cases, a hemizygous deletion or amplification of the *ALK* gene has been described [88]. *TFCP2*-RMS tumors are predominantly localized in the jaws and the skull of young adults, often with secondary involvement of soft tissues. The tumors are usually diagnosed at an advanced stage and have a rapid clinical course with a dismal prognosis and a 3-year overall survival of 28% despite aggressive multimodal therapy [89]. Treatment options include surgery which may not be indicated if massive tumor spread has occurred by the time of diagnosis. Chemotherapy and radiotherapy do not prolong survival. The combination of radiation and *ALK* inhibitors (crizotinib, alectinib, lorlatinib, and/or pazopanib) has shown partial response in anecdotal cases [90, 91].

### Adamantinoma-like Ewing sarcoma

Adamantinoma-like Ewing sarcoma (ALES) is a controversial variant of Ewing sarcoma (ES) defined by the presence of t(11;22) and *EWSR1::FLI1* fusion [92]. It is speculated whether it represents an epithelial or mesenchymal neoplasm, as epithelial markers (pan-cytokeratins and p63/p40) and ES-related markers (CD99 and NKX2.2) are commonly expressed [93]. It is uncertain whether this tumor should be managed according to carcinoma or sarcoma protocol. Many tumors are treated with surgery and adjuvant polychemotherapy according to ES-specific protocols.

### EWSR1/FUS::POU2AF3(COLCA2) sarcomas

**Fig. 6:**
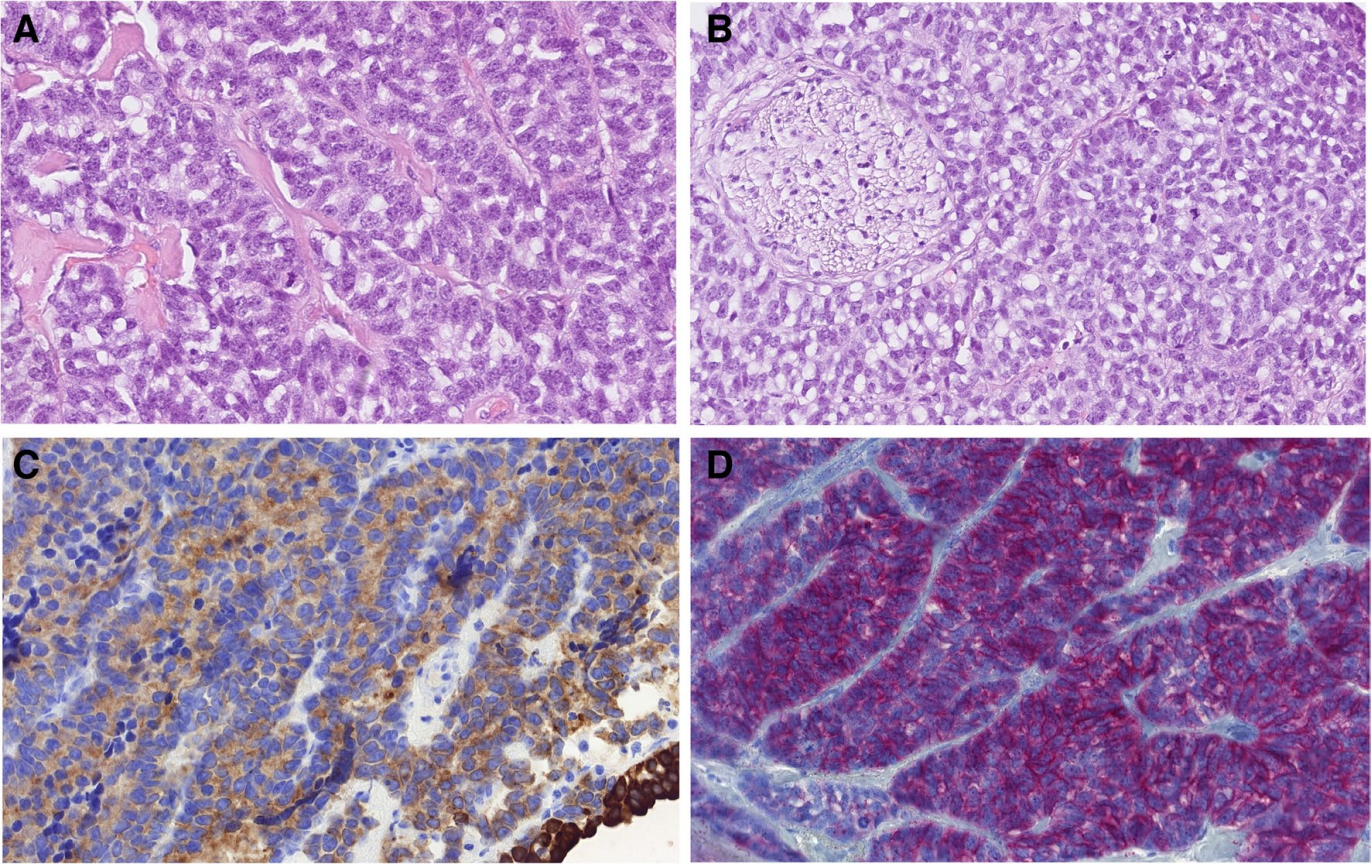
*EWSR1/FUS::POU2AF3(COLCA2)* sarcomas. The tumor consists of solid tumor nests with a neuroendocrine tumor-like appearance composed of basaloid tumor cells with scattered stroma (**A**). Extensive perineural spread is common (**B**). Weak OSCAR expression is occasionally present, the intensity of which can be compared with the strong expression of the mucosal epithelium (lower-right corner) (**C**). CD56 is an unspecific marker but is frequently expressed (**D**)

First reported by Agaimy et al. [94], *EWSR1/FUS::POU2AF3(COLCA2)* sarcomas are newly recognized aggressive neoplasms that exhibit a propensity to both local recurrence and metastatic spread despite multimodal treatment. They affect adult patients and commonly arise in the head and neck, with a striking predilection for the sinonasal tract. Their morphological spectrum ranges from spindle cell proliferation with mild nuclear atypia, through biphasic tumors composed of sheets and fascicles of spindled and round cells with features of neuroendocrine differentiation, to purely round cell tumors with high nuclear grade (Fig. 6A–D) [94–96]. In addition, some tumors were reported to contain foci of glandular, rhabdomyoblastic, or osteogenic differentiation [95]. Approximately half of the cases display pan-cytokeratin positivity [94–96], possibly leading to diagnostic confusion with synovial sarcoma. In general, *EWSR1* fusions with various partners are frequent in soft tissue tumors and include rare instances of alternative *EWSR1::SSX1* fusion in synovial sarcoma [97], or potential *FUS* fusion with *POU2AF3* instead of *EWSR1* [96]. Consequently, FISH detection of an *EWSR1* break is not a sufficient diagnostic test. Instead, targeted RNA sequencing is advised to render the correct diagnosis.

## Summary

In conclusion, with the widespread application of molecular testing, there has been a rapid development in the classification of head and neck tumors in general, but clearly also in the field of tumors of the sinonasal area and the anterior skull base. The availability of an expanding spectrum of immunohistochemical surrogates provides the pathologist with tools for rapid identification of diagnostic molecular abnormalities, that, for the clinicians, increasingly allow the selection of adequate targeted therapies. While still largely relying on histology and immunohistochemistry for diagnosis, the new insights into the molecular basis of sinonasal tumors clearly allow pathologists nowadays to increase their diagnostic accuracy, allowing for better fine-tuning and eliminating overlapping diagnoses. It is fair to say that the adoption of the molecular biological basis of sinonasal malignancies in the 5th edition of the WHO classification reflects the irreversible place these techniques have conquered in the daily practice of head and neck cancer departments.

## Data Availability

Data supporting the findings of this study are available within the article. The complete datasets generated during and/or analyzed during the current study are available from the corresponding author upon reasonable request.
